# Direct Medical Care Costs Associated With Patients Diagnosed With Chronic HCV

**DOI:** 10.5812/hepatmon.8415

**Published:** 2013-04-28

**Authors:** Sara Ashtari, Mohsen Vahedi, Mohammad Amin Pourhoseingholi, Maryam Karkhane, Zahra Kimiia, Asma Pourhoseingholi, Azadeh Safaee, Bijan Moghimi-Dehkordi, Mohammad Reza Zali, Seyed Moayed Alavian

**Affiliations:** 1 Gastroenterology and Liver Diseases Research Center, Shahid Beheshti University of Medical Science, Tehran, IR Iran; 2 Department of Epidemiology and Biostatistics, School of Public Health, Tehran University of Medical Sciences, Tehran, Iran; 3 Department of Disease Control and Prevention, Deputy of Health, Shahid Beheshti University of Medical Science, Tehran, IR Iran; 4 Baqiyatallah Research Center for Gastroenterology and Liver Diseases, Baqiyatallah University of Medical Sciences, Tehran, IR Iran

**Keywords:** Hepatitis C, Chronic, Health Care Costs, Peginterferon Alfa-2a

## Abstract

**Background:**

HCV virus (HCV) is a significant global problem with wide-ranging socio-economic impacts. Because of the high morbidity and mortality associated with end-stage liver disease, cirrhosis, and hepatocellular carcinoma (HCC), the economic burden of HCV infection is substantial.

**Objectives:**

This study aimed to estimate the direct medical care costs of chronic HCV infection.

**Patients and Methods:**

For this cross-sectional study, 365 courses of HCV treatment were extracted from medical records of 284 patients being referred to Tehran HCV clinic, a clinical clinic of Baqiyatallah Research Center for Gastroenterology and Liver diseases, from 2005 to 2010. All the patients had been diagnosed with HCV. Direct medical care costs for each course of HCV treatment have been calculated based on Purchasing Power Parity Dollar (PPP$).

**Results:**

Average direct medical costs for the courses treated with conventional interferon plus ribavirin (INF-RBV) were 4,403 PPP$, and 20,010 PPP$ for peg-interferon plus ribavirin (PEG-RBV) courses. There was an increase of the direct costs in both courses of treatment to achieve Sustain Viral Response (SVR). The costs amounted to 10,072 PPP$ in (INF-RBV) treatment and 34,035 PPP$ in (PEG-RBV). The significant difference between the costs of these two courses of treatment is attributable to high cost of Peg-interferon. This indicates that the medication costs are the dominant costs.

**Conclusions:**

According to the results, total direct medical costs for HCV patients in Iran exceeded 12 billion PPP$ in (INF-RBV) treatment and 55 billion PPP$ in (PEG-RBV).

## 1. Background

HCV infection is a global health problem affecting over 170-200 million people. The virus is distributed world-wide and its prevalence varies in different countries from 0.2% up to 40% (1-4). HCV prevalence has changed significantly world-wide, showing a decreasing trend in the developed world due to a decrease in infections among injecting drug users, the effect of harm reduction programs, and the reduced risk of transfusion-associated acute HCV. In contrast, HCV prevalence is high in undeveloped countries and high-risk groups (5). The prevalence of HCV infection in the general population is less than 1% in Iran (6, 7). In addition, HCV is one of the most common causes of chronic liver disease, and the third leading cause of death in patients with end-stage renal disease (8, 9). Patients with chronic HCV have a 15% risk of developing cirrhosis for an average period of 15 years, and 1-5% risk of developing HCC (10, 11). HCV places a significant burden on health care system. Burden of the disease, for both mortality and cost, is expected to increase over the next decade. HCV infection would be a potential cause of morbidity, mortality and liver transplantation in the future (1, 4, 12, 13). HCV can also place a high socio-economic burden on the individuals affected and the society. This disease can also become chronic and similar to other chronic diseases; it can impose enormous costs both on the patients as well as on health and treatment system (14, 15). Thus, it is not surprising that the health costs are the main concern of many health policy makers and academics in many countries (16, 17). Knowledge of the costs of an illness can help policy makers to decide which diseases need to be addressed first by health care and prevention policies. Cost-of-illness studies show the financial impact caused by a disease on public programs (18). It is always useful to measure economic burden and health care effectiveness to better understand and evaluate various intervention programs in the country. Despite some studies (19, 20) confirming high costs of HCV treatment and its importance on policy-making and health programs, in our country there are not available studies related to the economic burden of HCV. Therefore, performing such a study seems to be essential.

## 2. Objectives

The specific purpose of this study was to determine direct medical care costs of patients with chronic HCV who received the treatment of INF-RBV and PEG-RBV. Finally, the average direct medical care costs were calculated to achieve SVR.

## 3. Patients and Methods

### 3.1. Patient Selection

All the data for this cross-sectional study were collected from medical records of 284 patients with HCV who were referred to Tehran HCV clinic, a clinical clinic of Baqiyatallah Research Center for Gastroenterology and Liver diseases from 2005 to 2010. Therefore, a checklist was designed and developed. Based on this checklist, the information on the dosage and frequency of health services utilization and their related costs such as the fees of physician’s routine visits, confirmatory tests (endoscopy, ultrasound, liver biopsy, pathology, and electrophoresis), laboratory tests and diagnostic markers of hepatitis, hospitalization costs related to liver biopsy and drug costs were extracted from the patients’ records. The other variables which were included in the mentioned check list were personal information such as age, gender, profession, geographic region, urban/rural residence ,the patients’ socio-economic information, history of blood transfusion, addiction (IV drug user), needle, stick and some other risk factors. Finally, 365 courses of HCV treatment were extracted from the information found in the medical records of these 284 patients. All the 365 courses, which met the necessary requirements for the study, were the ones only used for the treatment of HCV, and there was no interference with the treatment of other diseases such as hemophilia, thalassemia, diabetes, hepatitis B, kidney disorders, and some other diseases. That is because, if a patient had HCV along with other diseases, the treatment course would differ and the treatment cost would differ accordingly.

### 3.2. Protocol of HCV Treatment

Antiviral therapy plays an important role in treating patients with HCV infection, because SVR prevents progression of fibrosis, decreases hepatic inflammation and necrosis, reduces the risk of HCC, and improves patient survival (5, 19, 20). Genotype, viral load and liver biopsy are important parameters used in selecting an antiviral therapy with the maximum chance of success (21, 22). In the past, the combination therapy of INF-RBV was considered as a gold standard (3 MIU thrice weekly along with ribavirin 800 to 1200 mg per day). This treatment enhances SVR rate up to 38-43%. Achieving SVR is greatly dependent upon HCV genotype; in a way that genotype 1 requires 48 weeks of treatment to achieve SVR of 29%, while genotypes 2, and 3 need up to 24 weeks of treatment to attain SVR rate of 66% (23, 24). Currently, the regular treatment of HCV is PEG-RBV (Alfa 2a in a fixed dose of 180 micrograms per week along with ribavirin 800 to 1200 mg per day). This therapy achieves SVR of about 50% for genotype 1, and 80% for genotypes2, and 3 (25, 26). But, in many developing countries like Iran, conventional INF-RBV is still used for treating HCV, mainly because of financial reasons. A total of 365 HCV treatment courses provided the necessary data for the study. In 183 (50.1%) courses, conventional Interferon (Roche Products Ltd) with ribavirin (Roche Products Ltd makes Copegus/MSD make Rebetol) had been used. In the remaining 182 (49.9%) courses, Peg-interferon (Roche Products Ltd make Peg-interferon alpha 2A: Pegasys) combination with ribavirin (Roche Products Ltd makes Copegus/MSD make Rebetol) had been used. Therefore, direct medical costs for both types of treatments were calculated separately, based on the relevant genotype.

### 3.3. Cost Analysis

The factors used for cost estimation included frequency of health resource utilization and unitary costs. Health resource utilization falls into the following categories including physician visit, hospitalization, confirmatory tests, laboratory tests and medication. Methodology of cost analysis in this paper is based on “Centers for Disease Control and Prevention” cost analysis introduction (27). Also, the cost calculation method used in this study is the same as the one used in other Iranian studies of the field (28-31). The unit cost of different health resources including physician (GP/specialist) visits, confirmatory and laboratory tests, and hospitalization was calculated based on the price lists approved by Iranian Cabinet for the Public Health Centers (32). And the price of drugs was retrieved from drug list of Food and Drug Office of Iranian Ministry of Health and Medical Education from 2005 to 2010 (33). Therefore, the unit cost of different health resources for each patient was calculated separately, based on their prices in different years (according to the year of treatment).Purchasing Power Parity Dollar (PPP$) was used to make intercountry comparisons. PPP$ is an economic technique used when attempting to determine the relative values of two currencies. It is useful, because the amount of goods a currency can purchase within two nations often varies drastically. It depends on availability of goods, demand for the goods, and a number other unknown factors. According to the reports released by Iranian Central Bank and World Bank Organization from 2005 to 2010 (34); for example, one PPP$ was estimated around 3,894 Rials in 2009. Therefore, 3,894 were used as the reference value to convert costs from Iranian Rials to PPP$.

### 3.4. Statistical Analysis

Descriptive statistics and frequency distributions such as mean, standard deviation, and percentage were used. T-test and one-way ANOVA were used to test the differences between means of continuous data. All statistical analyses were performed using SPSS, version 16.0 (SPSS Inc., Chicago. IL., USA). P < 0.05 was considered as statistically significant.

## 4. Results

**Figure 1.**
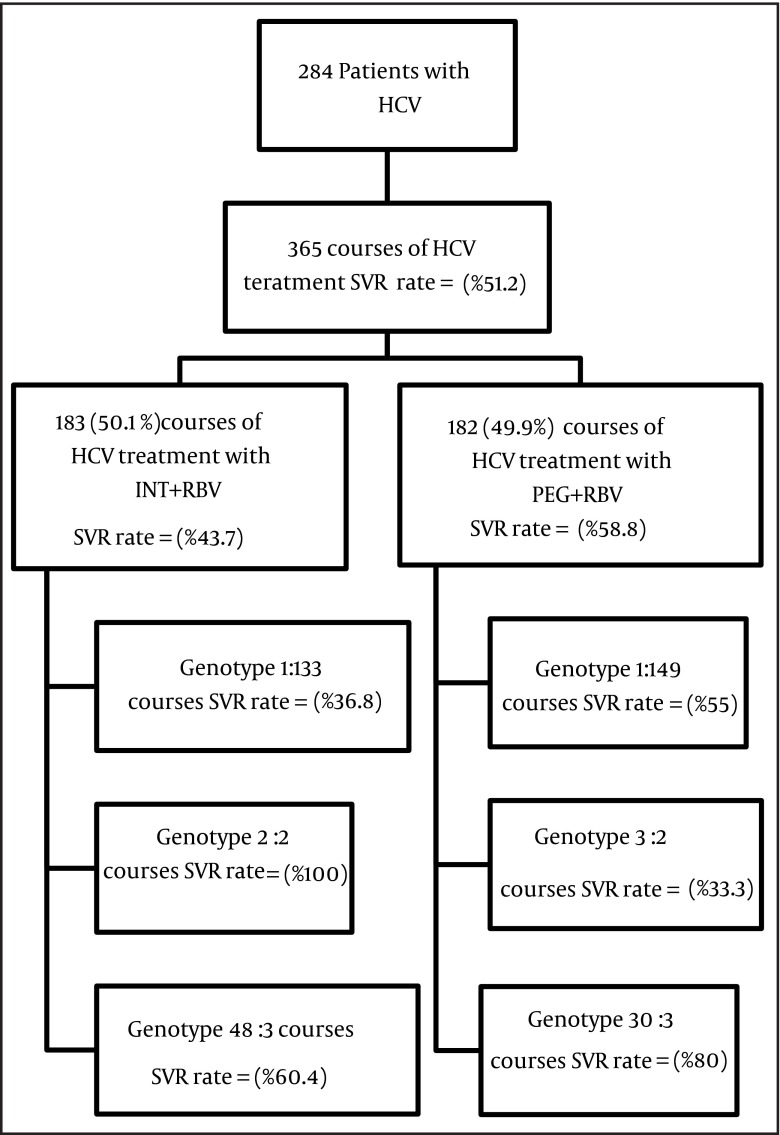
Overview of the Information and Finding of SVR Rate in This Study

In this study, 284 patients with the mean age of 41.6 ± 11.9 years had participated, of which 225 patients (79.2%) were male and 59 (20.8%) were female. Most patients (72.9%) were married, and education level of most (n = 142, 51.45%) was under high-school diploma. The highest Frequency of genotype 1a was (n = 179, 63%) followed by 3a (n = 64, 22.5%). Of the total number of patients, 87 (30.6%) had transfusion, 83(29.2%) had addiction, and 55 (19.4%) had needle stick history (Table 1). Average direct medical costs for INF-RBV and PEG-RBV courses were 4,403 PPP$ and 20,010 PPP$ respectively. Therefore, the average direct medical costs for PEG-RBV courses were significantly higher than that of INF-RBV (20.010 PPP$ vs. 4,403 PPP$; P < 0.001) (Table 2). Average costs of hospitalization (53 PPP$ vs. 40 PPP$; P = 0.012) and confirmatory tests (108 PPP$ vs. 80 PPP$; P < 0.001) in the courses of therapy with INF-RBV, were higher than those in the course of PEG-RBV. But the average costs of other categories such as physician visit (405 PPP$ vs. 365 PPP$; P = 0.030), laboratory tests (2,324 PPP$ vs. 1,979 PPP$; P < 0.001), and medication (17,160 PPP$ vs. 1,898 PPP$; P < 0.001), were higher in PEG-RBV course than in INF-RBV (Table 2). To achieve SVR, there was an in crease of direct medical costs in both treatment courses, 10,072 and 34,035 PPP$ for INF-RBV and PEG-RBV respectively (Table 3). The chance of achieving SVR in patients who had been treated with PEG-RBV was more than those treated with INF-RBV (OR = 1.837) (Table 4). Generally, in both therapeutic methods, the chance of attaining SVR is higher among the patients with genotype 3 than those with genotype 1 (Table 4). An overview of SVR rate and related information and findings has been presented in Figure 1.

**Table 1.** Demographic Characteristics of the Study Population (n = 284)

	Male, No. (%)	Female, No. (%)	Total, No. (%)
**Age group**			
14-35	79(36.7)	11(19.6)	90(33.2)
36-57	120(55.8)	37(66.1)	157(57.9)
58-79	16(7.5)	8(14.3)	24(8.9)
**Marital status**			
Single	59(26.7)	5(8.5)	64(22.9)
Married	154(69.7)	50(84.7)	204(72.9)
Divorced	8(3.6)	1(1.7)	9(3.2)
Widow	0(0)	3(5.1)	3(1.0)
**Education**			
Illiterate	46(21.2)	18(30.5)	64(23.2)
Under Diploma	110(50.7)	32(54.2)	142(51.4)
Diploma	42(19.4)	7(11.9)	49(17.8)
Bachelor and Upper	19(8.7)	2(3.4)	21(7.6)
**Genotype**			
1a	149(66.2)	30(50.8)	179(63.0)
1b	26(11.6)	10(16.9)	36(12.7)
2a	1(0.4)	0(0)	1(0.4)
2b	1(0.4)	2(3.4)	3(1.1)
3a	48(21.4)	16(27.2)	64(22.5)
3b	0(0)	1(1.7)	1(0.3)
**Transfusion**			
No	163(72.4)	34(57.6)	197(69.4)
Yes	62(27.6)	25(42.4)	87(30.6)
**Addiction**			
No	143(63.6)	58(98.3)	201(70.8)
Yes	82(36.4)	1(1.7)	83(29.2)
**Needle Stick**			
No	173(76.9)	56(94.9)	229(80.6)
Yes	52(23.1)	3(5.1)	55(19.4)

**Table 2.** Average Direct Medical Costs of HCV Treatment in Both Types of Combination Therapy (Costs are Expressed in PPP$)

Genotype	No.	Hospitalization, No.^a^	Confirmatory Tests, No.^b^	Physician Visit, No.^c^	Laboratory Tests^d^	Medication^e^	Total cost
** Interferon + Ribavirin **							
**1**	133	60	120	388	2,036	2,128	4,732
**2**	2	28	53	530	2,551	1,492	4,655
**3**	48	34	79	297	1,796	1,276	3,482
**Total**	183	53	108	365	1,979	1,898^f^	4,403^g^
** Peg + Ribavirin **							
**1**	149	43	83	422	2,384	18,582	21,515
**2**	3	41	121	464	2,435	14,838	17,899
**3**	30	27	68	311	2,013	10,327	12,745
**Total**	182	40	81	405	2,324	17,160^f^	20,010^g^
**Total**							
	365	46	95	385	2,151	9,508	12,185

^a^Short term of hospitalization due to liver biopsy

^b^Confirmatory tests include; Endoscopy, Sonography, liver biopsy, Pathology and Electrophorus

^c^Physician visit include; Costs of routine visits by a gastroenterologist

^d^Laboratory tests include; CBC, PT, FBS, TG, Chol, BUN, Cr, Bil.T, Bil.D, AST, ALT, ALP, T3, T4, TSH, Anti-TPO(ELISA), Anti-Thyroglobulin, T3Ru, HBsAg, HBsAb, HAVAb, HBcAb, Anti-HCV (ELISA), Anti-HCV (RIBA), Anti-HIV (ELISA), Genotype, PCR, Viral load

^e^Medication include; Costs of (INF-RBV) and (PEG-RBV)

^f^Statistical significant among medication costs of HCV treatment in both types of therapy was P<0.001

^g^Statistical significant among direct medical costs of HCV treatment in both types of therapy P<0.001

**Table 3.** Average Direct Medical Costs of HCV to Achieve SVR in Both Types of Combination Therapy (Costs are Expressed in PPP$)

Genotype	SVR^a^	Hospitalization, No.	Confirmatory Tests, No.	Physician Visit, No.	Laboratory Tests, No.	Medication	Total cost
** Interferon + Ribavirin **							
**1**	49	163	326	1,052	5,526	5,776	12,843
**2**	2	28	53	530	2,551	1,492	4,655
**3**	29	57	130	491	2,973	2,112	5,763
**Total**	80	121	248	836	4,526	4,341	10,072
** Peg + Ribavirin **							
**1**	82	78	151	768	4,332	33,765	39,094
**2**	1	124	364	1,392	7,304	44,513	53,697
**3**	24	33	84	389	2,517	12,908	15,932
**Total**	107	68	138	688	3,953	29,187	34,035
**Total**							
	187	91	185	752	4,198	18,558	23,784

^a^Abbreviations: SVR, Sustained Viral Responds

**Table 4.** Odds Ratio (OR) of SVR and 95% Confidence Interval to Medication and Genotype

Genotype	Non-SVR, No. (%)	SVR, No. (%)	Odd’s Ratio	95% CI for Odds’ Ratio	P value
** Interferon + Ribavirin **					0.021
**1**	84(81.6)	49(61.3)	1	-	
**2**	0(0)	2(2.4)	-	-	
**3**	19(18.4)	29(36.3)	2.617	1.329-5.151	
**Peg + Ribavirin**					0.036
**1**	67(89.3)	82(76.6)	1	-	
**2**	2(2.7)	1(.9)	0.409	0.036-4.604	
**3**	6(8)	24(22.5)	3.268	1.263-8.460	
** Interferon + Ribavirin **					0.004
	103(57.9)	80(42.8)	1	-	
** Peg + Ribavirin **					0.004
	75(42.1)	107(57.2)	1.837	1.213-2.782	

## 5. Discussion

In this study, it was found that the direct medical costs of HCV treatment with PEG-RBV are significantly higher than those courses treated with INF-RBV; that is undoubtedly, because of high costs of PEG. To achieve SVR, the direct medical costs would be definitely increased in both types of therapies. One reason could be that, fail to reach SVR and need to repeat the treatment course and this would increase the costs. Another reason might be related to the nonresponders patients who their antiviral regimen was stopped after one course of therapy (12 weeks or 24 weeks). According to the results, in PEG-RBV treatment, medication (85.7%) was found to be the dominant cost, while in INF-RBV courses the expenditure of laboratory tests (45%) was found to be the dominant cost. This is certainly because of the low cost of INF. And also the average costs of confirmatory tests and hospitalization were higher in INF-RBV. The treatment of HCV is very costly, and it is considered a high priority for health policy-makers, and especially for the patients infected with HCV genotype 1. Patients with HCV genotype 1 are considered as the ones difficult to treat. According to the study of Amini et al, the most common HCV genotype in Iran was genotype 1a, and after that genotypes 3a and 1b had the highest prevalence (35). This has also been approved by the findings of the present study. Genotype 1, in particular, cannot be treated efficiently with INF-RBV, while genotypes 2 and 3 respond favorably to the treatment. According to the results of this study, in PEG-RBV courses, the chance of achieving SVR in patients with genotype 3 is three times as much as the patients with genotype 1 (OR = 3.268). As for INF-RBV, the chance of achieving SVR in patients with genotype 3 is two times as much as those with genotype 1 (OR = 2.617). Moreover, genotype 1 infection may become chronic and more severe, and finally leads to cirrhosis and HCC much faster than genotypes 2 and 3 (21, 36). Based on the findings in this study, we had 18 (6.3%) patients with cirrhosis, and from these patients 16 (5.6%) had genotype 1, and 2 (0.7%) had genotype 3. In addition, the results indicate that the average direct costs for achieving SVR in each treatment course are higher in patients with HCV genotype 1 than those with other genotypes. Outcome of the first course of treatment showed that, of 36 patients who were resistance 29 (80.6%) patients had genotype 1 or 32 (82.1%) patients from 39 patients who had withdrawn from their treatment had genotype 1. And 37 (84.1%) from 44 patients and 16 (88.9%) from 18 patients who had relapse, and stopped their antiviral regimen according to their physician discretion had genotype 1. However the frequency of patients with genotype 1 is higher than patients with genotypes 2 and 3. Higher possibility of achieving SVR in PEG-RBV courses is another finding of this study, and it is quite compatible with the results obtained from the study performed by Fried et al, which showed that Peg-interferon combination therapy was more effective than Interferon combination therapy (25). Therefore, these findings are of high value and importance to health policy-makers in the country. Based on the available information on the direct medical care cost of a HCV treatment by two common therapeutic methods in Iran and the evidence gathered from a few population-based studies already performed in the country on prevalence of HCV, a rough estimate of direct medical care cost during one course of HCV can be estimated. According to the information of 6 provinces in 2009 the prevalence of HCV was reported to be 0.16% by Alavian et al (6). Since all the participants were from urban areas, it is assumed that our estimation of HCV cost per person only applies to urban adult population of Iran. According to the latest report published by the Statistical Center of Iran in 2006 (37), urban adult population was 17,218,066 million. The total direct cost of treating HCV was estimated to be 12 billion PPP$ with INF-RBV, and 55 billion PPP$ with PEG-RBV; assuming that the HCV prevalence, according to the existing studies, is 0.16%. The mentioned costs only cover the costs of adult urban population. Also these are the costs calculated only for one course of HCV therapy. It is clear that not every patient with HCV attains SVR by only one course of therapy; therefore, some of them may need another course of therapy, and this can lead to extra expenses for patients and the society. To better understand this it is noteworthy that, in this study from 284 patients with HCV 147 (51.8%) achieved SVR in the first course of therapy and the others; 39 (13.7%) patients withdrew from their therapy, 44 (15.5%) patients had relapse, 36 (12.7%) patients were resistance, and 18 (6.3%) stopped their antiviral regimen based on their physician discretion. So from these patients, 72 (25.4%) patients were entered the second phase of treatment and from these, 35 (48.6%) patients attained SVR. And finally 5 (55.6%) of 9 (3.2%) patients, who were entered the third phase of treatment achieved SVR. Thus the medical costs of HCV treatment would be increased with more courses of treatment. According to physician discretion, some patients may enter the second or third course of treatment, if the chance of achieving SVR exists for them. Nonresponders patients and patients with cirrhotic are not entered the second or third phase of treatment. Continuing the treatment for these patients is regarded as a waste of time and money. Because continuing the treatment for these patients does not lead to any improvement. And also the treatment strategy for patients with cirrhosis is different from other patients with HCV. So they entered different phase of treatment that this conflicts with our purpose in this study. Other group patients such as those who are resistant or stopped their treatment based on their physician discretion and who had relapse, may entered the second or third course of treatment unless they received different alternative treatment. For example, 22 patients from 36 patients who were resistant entered the second course of treatment, and in this course they treated with PEG-RBV instead of INF-RBV, and 9 (41%) patients from them attained SVR. Or 8 patients from 18 patients that were stopped their treatment according to their physician discretion, entered the second course of treatment, and 3 (37.5%) achieved SVR. Thus the rate of SVR in the second and third courses of treatment increased to 187 (65.8%). The study faced some limitations. First, this study was not population-based; therefore, the selection bias of the study population must be kept in mind. Second, the study was performed in the center of Tehran (Capital of Iran), and the participants were mostly from the urban areas. Third, the study only measured direct costs; thus, other indirect costs resulting from missed hours from work and school as well as transportation costs have not been taken into account. Finally, the number of times the patients have used the medical resources has been calculated using the patients’ records, which might be defective, and this could be also a source of bias. Regarding the obtained results, it is obvious that the direct medical costs of patients with HCV are so high. Future studies on the economic burden of HCV should attempt to estimate the indirect costs such as productivity loss, transportation, time spent by patients seeking care, costs incurred by caregivers and intangible costs such as emotional anxiety and fear, pain and stigmatization; so that the realistic and precise estimations of economic burden of HCV can be achieved.
